# Supplementary material for: Cryo-EM reveals a phosphorylated R-domain envelops the NBD1 catalytic domain in an ABC transporter
Source: Life Sci Alliance. 2024 Aug 29;7(11):e202402779. doi: 10.26508/lsa.202402779 (PMC11361370; doi:10.26508/lsa.202402779)
Supplement: Supplementary file 1 [file LSA-2024-02779_TableS1.docx]

**Table S1.** Ycf1 phosphorylation spectrum counts.

| P39109 – Metal resistance protein YCF1 OS=Saccharomyces cerevisiae (strain ATCC 204508 / S288c) GN=YCF1 PE=1 SV=2 | | | | | |
| --- | --- | --- | --- | --- | --- |
| Site | Modification | Best A-score | Localization Probability | Sample 1  100 ng_60 min | Sample 2  100 ng_60 min |
| S251 | Phosphorylation | 58.42 | 100 % | 7 | 2 |
| S846 | Phosphorylation | 27.76 | 100 % | 1 | 0 |
| S869 | Phosphorylation | 30.97 | 100 % | 4 | 1 |
| S873 | Phosphorylation | 26.20 | 100 % | 1 | 0 |
| S878 | Phosphorylation | 26.20 | 100 % | 8 | 4 |
| **S903** | **Phosphorylation** | **104.74** | **100 %** | **56** | **52** |
| S908 | Phosphorylation | 1,000.00 | 100 % | 13 | 56 |
| T911 | Phosphorylation | 1,000.00 | 100 % | 10 | 48 |
| S914 | Phosphorylation | 1,000.00 | 100 % | 10 | 52 |
| S1313 | Phosphorylation | 26.20 | 100 % | 0 | 1 |
| T1437 | Phosphorylation | 236.24 | 100 % | 1 | 1 |
