# Supplementary material for: Cryo-EM reveals a phosphorylated R-domain envelops the NBD1 catalytic domain in an ABC transporter
Source: Life Sci Alliance. 2024 Aug 29;7(11):e202402779. doi: 10.26508/lsa.202402779 (PMC11361370; doi:10.26508/lsa.202402779)
Supplement: Supplementary file 2 [file LSA-2024-02779_TableS2.docx]

**Table S2.** Cryo-EM data collection and refinement statistics

| **Data Collection** | |
| --- | --- |
| Microscope | ThermoFisher Titan Krios |
| Detector | Gatan K3 |
| Image pixel size | 0.822 Å |
| Defocus range | -0.9 to -2.1 µm |
| Electron exposure | ~ 55 electron/Å |
| Number of frames | 64 |
| Number of micrographs | 8,587 |
|  |  |
| **Image Processing** | |
|  | PKA IF-wide |
| No. of particles in final reconstruction | 68,169 |
| Symmetry | C1 |
| Final box size (pixels) | 440 |
| Global resolution (CRYOSPARC map) | 3.23 Å |
| FSC threshold | 0.143 |
|  |  |
| Refinement |  |
| Atoms | 23,726 |
| Residues | 1,481 |
| Water | 0 |
| Supplied Resolution | 3.23 Å |
|  |  |
| B-factor (Å^2^) |  |
| Iso/Aniso (#) | 11777/0 |
| Protein (min/max/mean) | 48.96/232.26/118.32 |
|  |  |
| Bonds (RMSD) |  |
| Length (Å) | 0.005 |
| Angles (°) | 0.889 |
|  |  |
| **Validation** | |
| MolProbity score | 0.62 |
| Clash score | 0.34 |
| Ramachandran plot (%) |  |
| Outliners | 0 |
| Allowed | 1.30 |
| Favored | 98.70 |
| Rotamer outliners (%) | 0 |
|  |  |
| **Model vs Data** | |
| CC (mask) | 0.84 |
| CC (box) | 0.65 |
| CC (peaks) | 0.50 |
| CC (volume) | 0.83 |
|  |  |
| PDB ID | 9YAC |
| EMDB ID | EMD-43985 |
